# Comparative Cytotoxicity of Artemisinin and Cisplatin and Their Interactions with Chlorogenic Acids in MCF7 Breast Cancer Cells

**DOI:** 10.1002/cmdc.201402285

**Published:** 2014-09-10

**Authors:** John O Suberu, Isolda Romero-Canelón, Neil Sullivan, Alexei A Lapkin, Guy C Barker

**Affiliations:** [a] School of Life Sciences, University of WarwickCV4 7AL (UK) E-mail: Guy.Barker@warwick.ac.uk; [b] Chemical Engineering and Biotechnology, University of CambridgeCB2 3RA (UK); [c] Department of Chemistry, University of WarwickCV4 7AL (UK); [d] SensaPharm Ltd., 123i Bioscience CentreSunderland, SR5 2TA (UK)

**Keywords:** antagonism, *Artemisia* tea, artemisinin, chlorogenic acid, cisplatin, synergy

## Abstract

In parts of Africa and Asia, self-medication with a hot water infusion of *Artemisia annua* (*Artemisia* tea) is a common practice for a number of ailments including malaria and cancer. In our earlier work, such an extract showed better potency than artemisinin alone against both chloroquine-sensitive and -resistant parasites. In this study, in vitro tests of the infusion in MCF7 cells showed high IC_50_ values (>200 μm). The combination of artemisinin and 3-caffeoylquinic acid (3CA), two major components in the extract, was strongly antagonistic and gave a near total loss of cytotoxicity for artemisinin. We observed that the interaction of 3CAs with another cytotoxic compound, cisplatin, showed potentiation of activity by 2.5-fold. The chelation of cellular iron by 3CA is hypothesized as a possible explanation for the loss of artemisinin activity.

## Introduction

Cancer is a major public health problem with about 7.6 million deaths in 2008; this number is projected to increase to over 13 million in 2030.[1] Although a range of treatment options are available, in many cases these therapies are fraught with significant levels of toxicity to healthy cells, and drug resistance quickly develops in some treatment regimes. To decrease the current cancer burden, drug discovery is directed at the development of highly effective and potent medications with reduced side effects.

**Figure 1:**
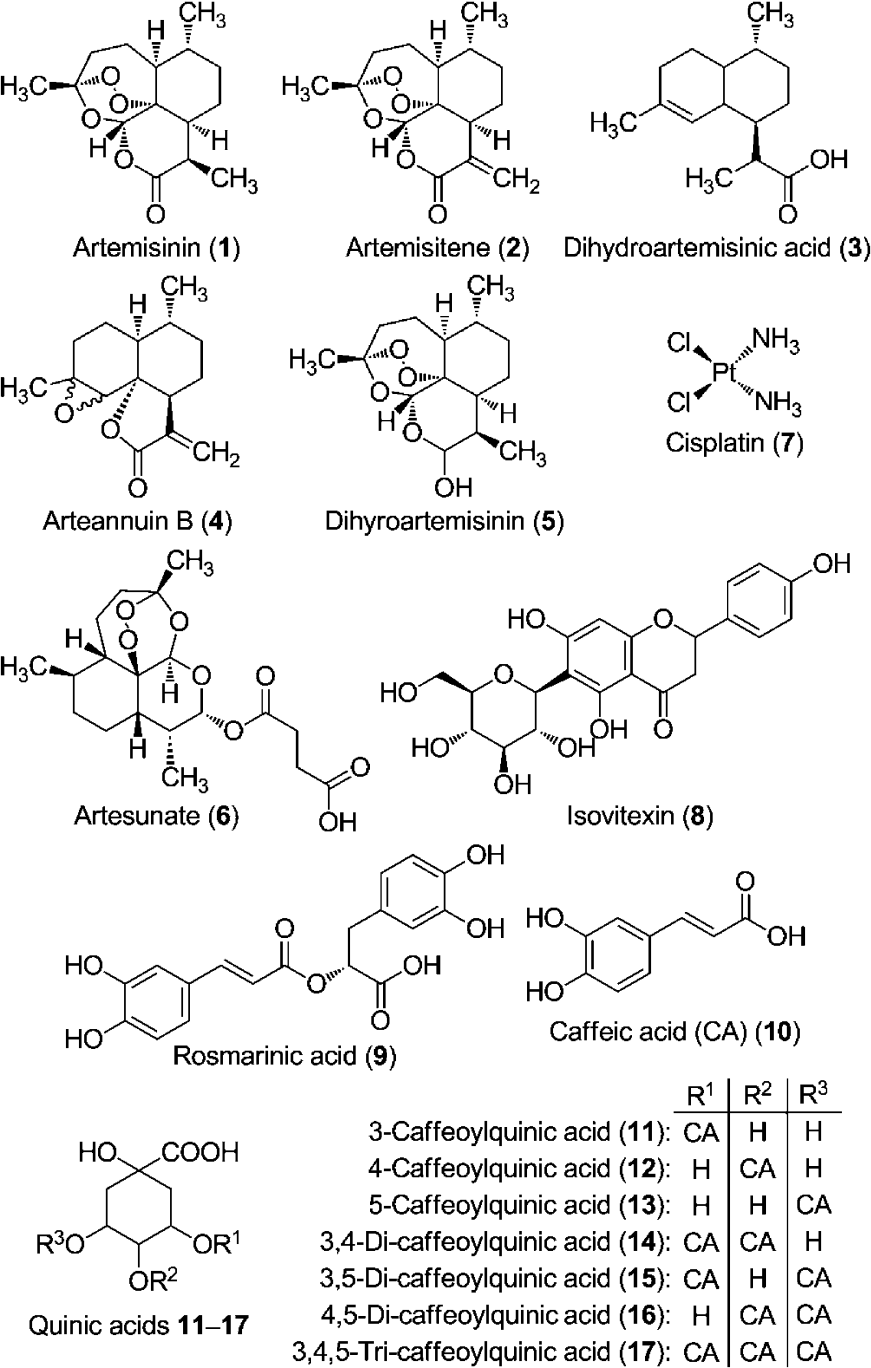
Structures of some compounds found in *Artemisia* aqueous extract (tea) and cisplatin.

Natural products are a rich source of active principles against cancer cells. A major success of pharmacognosy is the isolation of paclitaxel (Taxol) from the bark of the Pacific yew tree (*Taxus brevifola*). Paclitaxel exerts its anticancer effect by inhibiting mitosis and is now a drug approved by the US Food and Drug Administration (FDA) for ovarian and breast cancers.[2] Another success is the antimalarial compound artemisinin (**1**), a sesquiterpene lactone derived from *Artemisia annua* L. that possesses a unique trioxane bridge (Figure 1).

The in vitro cytotoxic activity of artemisinin and its derivatives has been reported in various cancer cell lines, including drug-resistant lines.[3] Sigh and Lai showed that a combination of dihydroartemisinin (**5**) and holotransfferin effectively kills radiation-resistant breast cancer cells,[3b] while artemisinin pretreated with holotransfferin was also found to be effective toward both drug-sensitive and multi-drug-resistant human lung carcinoma (SCLC) cells.[3c] Artesunate (**6**) inhibited the growth of highly angiogenic Kaposi sarcoma cells, showing the anti-angiogenesis effect of artemisinins.[4] In their study, Chen et al. implanted nude mice with human ovarian cancer cells and found that artesunate decreased tumor growth and significantly lowered vascular endothelial growth factor (VEGF) expression in the cells.[5] The potential of artemisinin to prevent the development of breast cancer in rats treated with a known carcinogen (7,12-dimethylbenz[*a*]anthracene, DMBA) has been reported.[6] Artesunate has also been successfully used in combination with standard chemotherapy to treat metastatic melanoma in human subjects after standard chemotherapy alone was ineffective in stopping tumor growth.[7]

Artemisinin and its derivatives have also been used as chemosensitizers for conventional treatments in drug-resistant cancer cell lines.[8] Synergistic interaction of dihydroartemisinin with gemcitabine, a cancer drug, showed a 45 % enhancement in tumor growth inhibition compared with the drug alone.[9] The improved efficacy of multicomponent combinations involving artemisinin in cancer treatment has encouraged investigation of other natural compounds besides artemisinin that may exhibit individual cytotoxic activity or that can be potential artemisinin synergists in the crude extract. Two artemisinin-related compounds, artemisitene (**2**) and arteannuin B (**4**), and two unrelated ones, scopoletin and 1,8-cineole, have shown antiproliferative activity.[10] No cross-resistance to artemisinin was observed with any of these actives, thus showing a potential for use in combination to treat drug-resistant tumors.

In the artemisinin research community, a significant degree of interest has been focused on the activity of aqueous extracts (*Artemisia* tea).[11] This interest stems largely from the widely reported use of *Artemisia* aqueous infusions in folk medicine. Through in vitro tests, we recently showed that some constituents of the extract interact synergistically with artemisinin, resulting in increased antiplasmodial activity.[12] Consequently, we were interested in the interactions of artemisinin with co-metabolites in the extract, in view of improved cytotoxicity.

Carbonara et al. observed that the major constituents of *Artemisia* tea are chlorogenic acids (**11**–**17**).[13] They also detected a number of feruloylquinic acids together with some flavonoids in the extract. Chlorogenic or caffeoylquinic acids (CQAs) are esters of caffeic (**10**) and quinic acids. The pharmacological properties of these catechols include antioxidant, hepatoprotectant, antibacterial, antihistiminic, chemopreventive, and other biological effects.[14] Lee and Zhu showed that chlorogenic acids and other catechol-containing dietary polyphenols can inhibit the methylation of synthetic DNA substrates in vitro and can inhibit the methylation of the promoter region of the RAβ gene in human breast cancer cells; both are normally hypermethylated in neoplastic cells.[15] In their study, Noratto et al. showed the chemopreventive potential of dietary chlorogenic and neochlorogenic acids.[16] These compounds exerted relatively high growth inhibition on the estrogen-independent breast cancer cell line and low toxicity in normal cells. Chlorogenic acid derivatives were also found to inhibit hepatocellular carcinoma cell line proliferation and induced apoptosis in leukemia cell lines.[17]

This study was therefore undertaken to evaluate the in vitro cytotoxicity toward breast cancer cells of *Artemisia* tea and artemisinin in combination with co-metabolites present in the tea extract. It specifically looks at the interaction of artemisinin with chlorogenic acid (3-caffeoylquinic acid, 3CA) to assess possible implications for the use of *Artemisia* tea in cancer therapy and compares this with cisplatin’s interaction with 3CA in MCF7 cells.

## Results and Discussion

The metabolite profile and cytotoxic activity of *Artemisia* hot water infusion (tea) in MCF7 breast cancer cells was evaluated and compared with the activity of artemisinin alone and in combination with chlorogenic acid, a co-metabolite in the extract. A combination study of chlorogenic acid with the drug cisplatin [*cis*-diamminedichloroplatinum(II)] was carried out for comparative analysis and possible elucidation of the interactions in *Artemisia* tea extract.

### Composition of *Artemisia* tea

The profile of metabolites in aqueous extract is listed in Table 1. These were analyzed by both MS–MS and HPLC methods. The profiling is based on the extensive analysis by Carbonara et al.[13] and on our earlier work[18] with artemisinin-related compounds in the extracts.

**Table 1:** Metabolites in the aqueous *Artemisia* extract analyzed by both MS–MS and HPLC methods

Compound	Amount [mg (l tea)^−1^]^[a]^
artemisinin	47.50±0.80
arteannuin B	1.30±0.01
caffeic acid	0.80±0.03
3,5-dicaffeoylquinic acid	57.00±1.70
3-caffeoylquinic acid	72.00±1.60
4-caffeoylquinic acid	20.40±1.60
4,5-dicaffeoylquinic acid	31.60±4.00
5-caffeoylquinic acid	9.00±0.70
isovitexin	65.00±7.20
rosmarinic acid	1.10±0.01

[a] Values are the mean±SD of *n*=2 determinations of triplicate measurements.

The levels of artemisinin reported in the tea extracts are varied and the values obtained in this study (47.5±0.8 mg l^−1^) are within that range. This corresponds to about 16 % of clinical exposure to the drug in reported treatment regimes, which also showed that the bioavailability of artemisinin in tea is very similar to pure artemisinin administered as a capsule.[13, 19] Van der Kooy and Verpoorte[19a] have shown that the method employed in preparing the hot water infusion does affect the amount of artemisinin and other co-metabolites extracted. This study, as well as others,[19a, b] used the therapeutically recommended ratio of 1:200 *w*/*v* or 5 g l^−1^.[20] Arteannuin B (**5**) (1.3 mg l^−1^), a biosynthetic precursor of artemisinin, was also detected in the tea extract using our method.[18]

The most abundant of the caffeic derivatives **11**–**17** was 3-caffeoylquinic acid (**11**) (72 mg l^−1^) in the analyzed extract, followed by 3,5-dicaffeoylquinic acid (**15**) (57 mg l^−1^). A comparatively lower amount (0.8 mg l^−1^) was observed for caffeic acid (**10**). The only flavonoid analyzed was isovitexin (**8**) (65 mg l^−1^) and was relatively abundant in our extract. Rosmarinic acid (**9**) was lower (1.1 mg l^−1^) in our samples than the levels found by de Magalhães and co-workers.[19c]

### Cytotoxicity of cisplatin, artemisinin, and 3CA

Table 2 shows the 50 % inhibitory concentration for artemisinin, cisplatin and 3CA in MCF7 breast cancer cells. This cell line is derived from breast adenocarcinoma tissues and is a common model employed in carcinogenesis and chemopreventive studies.[21]

**Table 2:** IC_50_ values for artemisinin, cisplatin and 3CA in MCF7 cells

Compound	IC_50_ [μm]^[a]^
artemisinin	9.13±0.07
cisplatin	5.75±0.02
3-caffeoylquinic acid	126.98±0.13

[a] Values are the mean±SD of *n*=2 determinations of triplicate measurements.

#### Cytotoxicity of artemisinin

**Figure 2:**
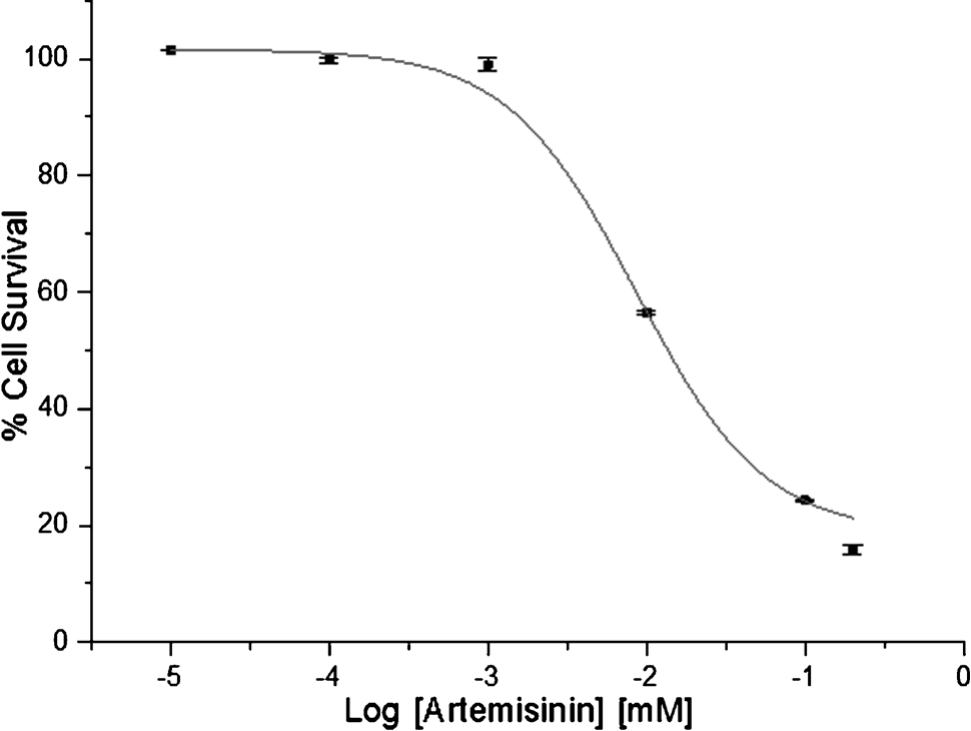
A dose–response curve for artemisinin in MCF7 cells. Percentage cell survival is plotted against the logarithm of treatment concentrations. Data points are the means±SD of duplicate determinations of triplicate measurements.

The cytotoxicity of artemisinin (Table 2 and Figure 2) in the MCF7 cells shows it to be potent against invasive breast ductal carcinoma that is estrogen sensitive. The IC_50_ values obtained for the compound (9.13±0.07 μm) are within a range of values (IC_50_ 0.17–87.10 μm) reported by Efferth and Oesch for artemisinin and its derivatives determined for the tumor panel of 60 cell lines of the National Cancer Institute (NCI) screening program.[22] Artemisinin had the highest IC_50_ value (least potent) of all the related derivatives reported. Artemisinin is metabolized into dihydroartemisinin (DHA), which has a lower IC_50_ value (2.3 μm) in MCF7 cells[22, 23]

Several researchers have investigated the mechanism of selective cytotoxicity of artemisinin and its derivatives toward neoplastic cells. Mercer et al. showed that selective activation of the trioxane bridge via carbon-centered radicals occurs in rapidly dividing or susceptible cells.[24] This then results in mitochondrial membrane depolarization, leading to induction of apoptosis by the chemical stress pathway and activation of caspases-3 and -7 in HL-60 cells, resulting in degraded DNA or hypodiploidy. Li et al. also showed that artemisinin derivatives induce apoptosis mainly through G_1_ arrest.[25] The G_1_ phase is associated with increased iron intake and transfferin receptor expression. Down-regulation of anti-apoptotic Bcl-2 proteins and up-regulation of pro-apoptotic Bax proteins have been associated with artesunate-treated human vein endothelial cells. Artemisinins have also been associated with lowered vascular endothelial growth factor (VEGF) expression. VEGFs are potent angiogenic factors.[26] These studies suggest that the mechanism(s) for the cytotoxicity of artemisinins involves many different pathways.

#### Activity of cisplatin

**Figure 3:**
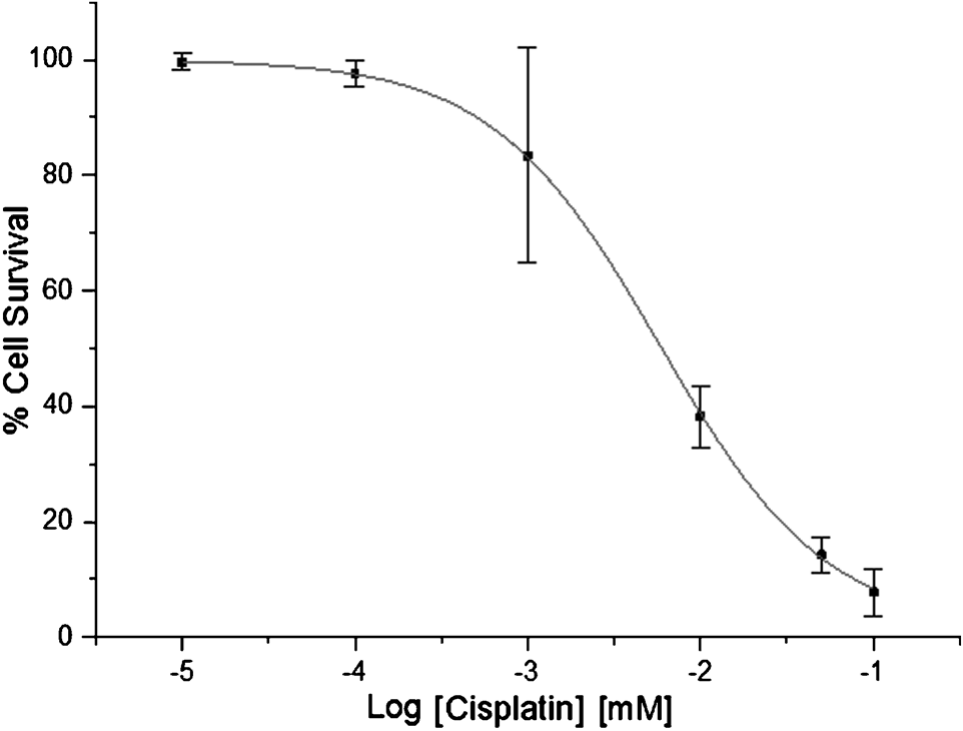
An IC_50_ curve for MCF7 cells treated with cisplatin. Percentage cell survival is plotted against the logarithm of treatment concentrations. Data points are the means±SD of duplicate determinations of triplicate measurements.

Cisplatin showed superior cytotoxicity in MCF7 cells compared with artemisinin (Table 2 and Figure 3). The mean IC_50_ value obtained (5.75±0.07 μm) is similar to values reported by Isikdag et al. (IC_50_ 8.6 μm) using MCF7 cells and the same duration of drug exposure.[27] Although cisplatin is very effective with solid-type carcinoma, drug resistance and toxic side effects have also been reported.[28]

As a platinum-based drug, cisplatin (**7**) exerts its cytotoxic effect through multiple mechanisms of which the most important and the best understood involves interaction with DNA to form GG intrastrand DNA cross-links, leading to the activation of several signal transduction pathways and culminating in the induction of mitochondrial apoptosis.[29] Consistent initial responses have been obtained by cisplatin treatment. However, these often result in the development of chemoresistance and therapeutic failure.[30] The combination of cisplatin with a chemosensitizer or a synergist can potentially improve efficacy and restore sensitivity to cisplatin.[31]

#### Cytotoxicity of 3CA

**Figure 4:**
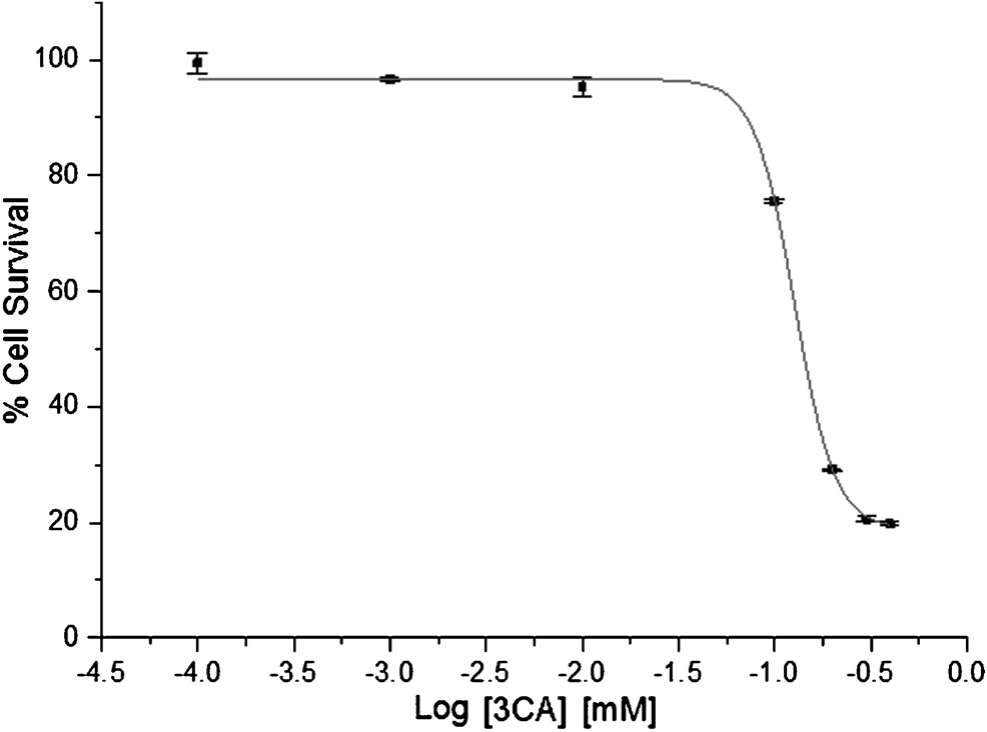
An IC_50_ curve for 3CA in MCF-7 cells. Percentage cell survival is plotted against the logarithm of treatment concentrations. Data points are the means±SD of duplicate determinations of triplicate measurements.

The IC_50_ for 3CA in MCF-7 cells (127.0±0.8 μm) was highest among the three single agents tested (Figure 4). This is similar to the observation by Lee et al., who reported that the growth inhibition of MCF-7 cells by 3CA was insignificant up to 20 μm and only inhibited by about 15 % at 50 μm concentration.[15a] Therefore, 50 % growth inhibition at a concentration of 126.9±0.1 μm, which we obtained, is in the range of the reported values. The chemopreventive and antiproliferation effects of 3-caffeoylquinic acid along with other dietary derivatives have also been reported.[16, 17]

The cytotoxicity of 3CA is dose dependent and is observable only above a certain concentration.[15a] Controversial and conflicting experimental results have been observed[32] in trials involving endogenous antioxidants such as 3CA because of their “double-edged sword” effect at cellular redox sites. Depending on the dosage level and the in situ matrix, these compounds can either be pro-oxidative or antioxidative.

### Cytotoxicity of *Artemisia* tea

**Figure 5:**
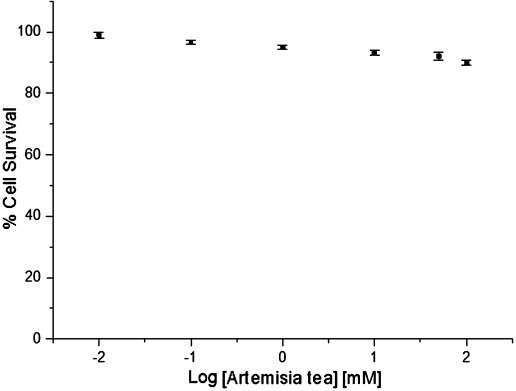
A dose–response curve for *Artemisia* tea extract in MCF7 cells. Percentage cell survival is plotted against the logarithm of treatment concentrations. Data points are the means±SD of duplicate determinations of triplicate measurements.

The dose–response for *Artemisia* tea is shown in Figure 5. Several repeat analyses gave values above 200 μm. The high IC_50_ values observed with tea and the complex composition of the same necessitated examination of the interactions among fewer metabolites. The combination of artemisinin with 3CA was subsequently investigated. In our analysis of the tea, 3CA was one of the most abundant components, second only to isovitexin (Table 1). Our choice of 3CA was also informed by its prominent dietary profile.

### Cytotoxic combination studies

The cytotoxicity of *Artemisia* tea was investigated to assess its possible role in cancer therapy. The unexpectedly high IC_50_ value for the phyto complex led to the investigation of simpler combinations in the extract. The combination of artemisinin and 3CA resulted in a drastic modification of artemisinin’s activity (Table 3). Consequently, we investigated the interaction of 3CA with cisplatin to see if a similar effect is reproduced in another anticancer drug.

**Table 3:** IC_50_ values for 3CA in combination with artemisinin and cisplatin in MCF7 cells

Compounds	IC_50_ [μm]^[a]^
artemisinin+3CA	>200
cisplatin+3CA	2.27±0.06

[a] Values are the mean±SD of *n*=2 determinations of triplicate measurements.

The combination of caffeic and chlorogenic acids with chemotherapeutic agents as chemosensitisers has been reported. An increased sensitivity of multidrug-resistant breast cancer cells (MCF-7/Dox) to doxorubicin was observed with caffeic acid.[33] A US patent for the use of chlorogenic acid as a sensitizer for chemotherapeutic agents reported a 30 % decrease in the viability of cancer cells sensitized by chlorogenic acids to doxorubicin compared with cells administered with doxorubicin alone.[34]

#### Artemisinin combination with 3CA

**Figure 6:**
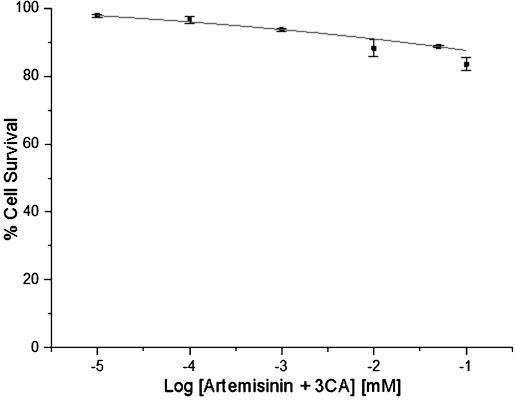
A dose–response curve for MCF-7 cells treated with a combination of artemisinin and 3CA. Percentage cell survival is plotted against the logarithm of treatment concentrations. Data points are the means±SD of duplicate determinations of triplicate measurements.

The dose–response curve for the combination of artemisinin and 3CA at a 1:1 molar ratio is shown in Figure 6. The IC_50_ in MCF-7 cells for the combination is >200 μm. This represents a near complete loss of cytotoxicity for the compound in the presence of 3CA (Table 2). A similar loss of activity was observed in combinations involving lower 3CA concentrations with artemisinin (artemisinin/3CA, 1:0.5 and 1:0.01). This suggests an antagonistic interaction between artemisinin and 3CA when combined and may partly explain the high IC_50_ values observed.

#### Cisplatin combination with 3CA

**Figure 7:**
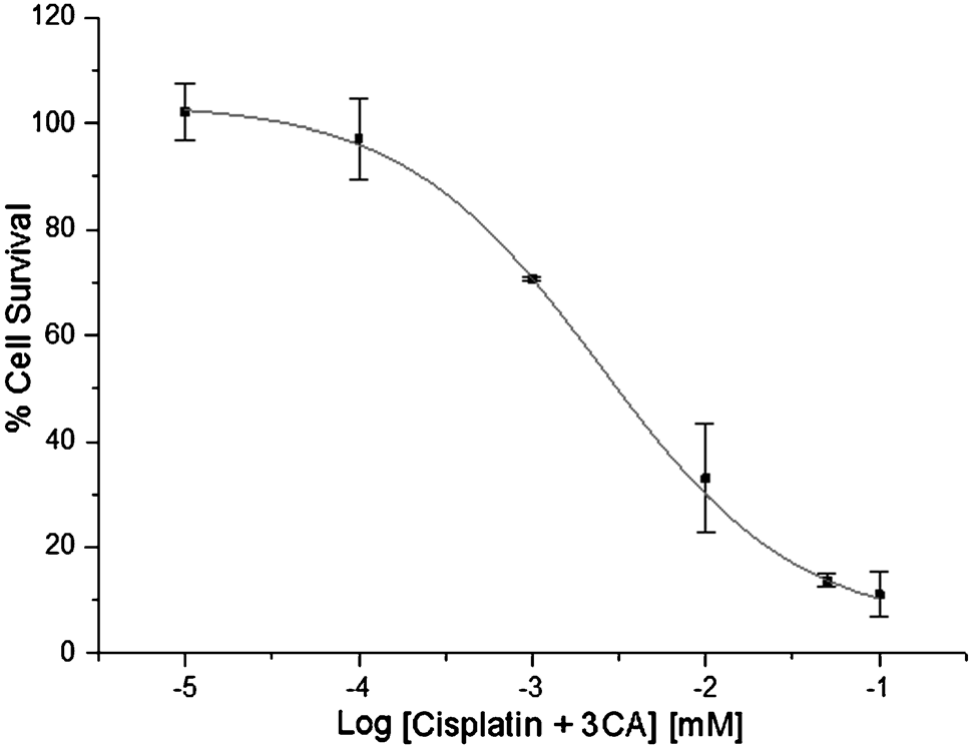
A dose–response curve for an equimolar combination of cisplatin and 3CA. Percentage cell survival is plotted against the logarithm of treatment concentrations. Data points are the means±SD of duplicate determinations of triplicate measurements.

To investigate if the strong antagonistic interaction observed for artemisinin and 3CA is reproduced in other anticancer agents, an equimolar combination of cisplatin and 3CA was tested. An IC_50_ value of 3.6 μm was obtained from the dose– response curve (Figure 7). This represents a 2.5-fold improvement in potency relative to cisplatin alone (Table 2). Kim reported the chemosensitizing effect of chlorogenic acids, and an improvement in activity of doxorubicin was observed when combined with chlorogenic acid in a range of combinations.[34]

The mechanism for the interaction of 3CA in MCF-7 with artemisinin (antagonism) and cisplatin (potentiation) seem to be pharmacokinetic in nature, where the observed change in activity for a combination relative to the single agent is due to modification in the absorption, distribution, metabolism, or excretion of a compound (cisplatin and artemisinin) by another (3CA).[35] The improvement in cytotoxicity of the combination of cisplatin and 3CA over cisplatin alone is less likely due to 3CA cytotoxicity, which is shown to be relatively inactive (Table 2).

The activation of artemisinin and the cleavage of the endoperoxide bridge to form a carbon-centered radical and/or reactive oxygen species (ROS) is a key to the compound’s cytotoxicity and antiplasmodial activities. This activation has been suggested to be initiated by endogenous iron, which is relatively abundant in actively dividing cells relative to normal cells.[3d] Kono et al. and others reported that 3CA has iron-chelating properties and forms a complex with the metal.[36] In a combination with artemisinin, 3CA may chelate and complex with endogenous iron, and as a result depletes the iron pool available for the activation of artemisinin. This effect will be more pronounced in the cytotoxic activity of artemisinin relative to its antiplasmodial activity, because the erythrocytic iron pool is severalfold more abundant than the neoplastic cell iron pool.[36a]

This is consistent with our observations for the combination in both antiplasmodial and cytotoxic assays. In the previous work, a mild antagonism was observed for the antiplasmodial activity of 3CA and artemisinin combination.[12] In the above cytotoxicity assay, strong antagonism (or a near total loss of activity) was observed (Figure 6). Williamson has reviewed similar adverse reactions (ADRs) in herbals and the methods by which antagonism arises in these mixtures.[37]

In contrast, cisplatin is activated in the cell by aquation of the molecule, resulting in the loss of one or both of its chloride ions. The activation is enhanced by a lower intracellular chloride ion concentration than its extracellular concentration.[28b] Metal ions do not seem to play any role in cisplatin activation and are thus unaffected by the metal-chelating properties of 3CA. It would be interesting to investigate whether cisplatin can chelate with CO_2_^−^ and deprotonated OH (six-membered ring) of 3CA.

## Conclusions

This study investigated in vitro the use of *Artemisia* tea as a chemotherapeutic agent using MCF7 cells. The high IC_50_ value observed for the tea extract led to the investigation of the combinations of 3-caffeoylquinic acid (3CA), a major component of the tea, with artemisinin, the main active ingredient in the extract. The combination showed a near total loss (strong antagonism) of cytotoxicity. This was in contrast to a 2.5-fold improvement observed when 3CA was combined with cisplatin, another anticancer agent. An explanation was suggested for these observations and also a possible reason was advanced for the difference in antiplasmodial and cytotoxicity of 3CA combination with artemisinin via endogenous iron-mediated activation of the artemisinin molecule.

Based on these results, the use of *Artemisia* tea in cancer therapeutics seems at best unpredictable and at worst ineffective. Further in vivo and in vitro investigations of the interactions between artemisinin with 3CA and other dietary antioxidants is imperative before any recommendation for the use of artemisinin and its derivatives as antiproliferative drugs with the possible avoidance of antioxidant food and drink immediately before and after intake of the drugs in single or combination therapies can be established.

## Experimental Section

**Chemicals**: Artemisinin (98 %), dimethyl sulfoxide (DMSO), chlorogenic acids, trichloroacetic acid (≥99 %), sulforhodamine B (SRB; 75 %), sodium phosphate monobasic monohydrate (≥99 %), sodium phosphate dibasic heptahydrate (≥99 %), acetic acid (≥99 %), and cisplatin were obtained from Sigma–Aldrich (Dorset, UK). Arteannuin B was gifted by Walter Reed Army Institute of Research (WRAIR) USA. LC–MS-grade formic acid in water, acetonitrile, and HPLC-grade acetonitrile were obtained from Fisher Scientific, UK. Purified water (∼18 MΩ cm^−1^) was dispensed from a Milli Q system (Millipore, UK). For the in vitro assays, RPMI 1640 medium, as well as fetal bovine serum, l-glutamine, a penicillin/streptomycin mixture, trypsin, and phosphate-buﬀered saline (PBS) were purchased from PAA Laboratories GmbH (Germany).

**Plant materials**: High-yielding dried *A. annua* biomass was obtained from BIONEX Madagascar and stored under dark, cool conditions until use.

**Plant extracts**: *Artemisia* tea was prepared according to published methods with a slight modification.[11a, 38] Briefly, 1 L of boiling water was added to 5 g of dried plant material, stirred and stored in the dark for 1 h. The extract was filtered in vacuo and lyophilized after freezing to obtain the dried tea extract. The ethanolic extract was obtained by sonication for 30 min in ethanol at 1:10 (*w*/*v*) biomass-to-solvent ratio. The sonication bath was kept cool with ice, and the extract was filtered and concentrated in vacuo at 30 °C, and further dried under a gentle stream of nitrogen gas. These extracts were used in the antiproliferation assays.

**MS–MS method for artemisinins**: The MS–MS method is described in detail elsewhere.[18] Briefly, the MS–MS system was operated with an ESI interface in positive ionization mode (ESI+). The cone and desolvation gas flow rates were set at 45 and 800 L h^−1^, respectively. MS parameters were automatically defined using Waters IntelliStart software for the tuning and calibration of the TQD and subsequently manually optimized for all analytes. Capillary voltage was set at 2.8 kV, collision voltage at 7 V, source temperature was 150 °C, and cone voltage was set at 24 V. A multiple reaction-monitoring (MRM) transition of 283→219+229+247+265 was used for artemisinin. Quantification was determined by using MRM modes for the above transitions. The dwell time was automatically set at 0.161 s. Data were acquired by MassLynx ver. 4.1 software and processed for quantification with QuanLynx ver. 4.1 (Waters Corp., Milford, MA, USA).

The HPLC system consisted of a binary pump, a cooling autosampler with an injection loop of 10 μL set at 10 °C. The column heater was set at 30 °C and a GenesisLightn C_18_ column (100×2.1 mm, 4 μm) (Grace, IL, USA) protected by an Acquity-LC column in-line filter unit (0.2 μm in-line frit) was used for separation of metabolites. The mobile phase consisted of A: 0.1 % formic acid in water and B: 0.1 % formic acid in acetonitrile used in the following gradient: 0–7.00 min, 25→98 % B; 7–9.5 min, 98 % B; 9.5–10 min, 98→25 % B; 10–15 min, 25 % B at a flow rate of 0.4 mL min^−1^. Weak wash solvent was 10 % acetonitrile, strong and needle wash solvent was a mixture of acetonitrile, propan-2-ol, methanol, and water (30:30:30:10 *v*/*v*/*v*/*v*).

**Cell culture**: MCF7 human breast carcinoma cells were obtained from the European Collection of Cell Cultures (ECACC) and used between passages 5 and 18. The cells were grown in RPMI 1640 supplemented with 10 % fetal calf serum, 1 % 2 mm l-glutamine, and 1 % penicillin/streptomycin, as adherent monolayers at 310 K in a 5 % CO_2_ humidified atmosphere and passaged at approximately 70–80 % confluence.

**In vitro growth inhibition assays**: Briefly, 5000 cells were seeded per well in 96-well plates. The cells were pre-incubated in drug-free media at 310 K for 48 h before adding various concentrations of the compounds to be tested. Stock solutions of the compounds were first prepared in 5 % DMSO and a mixture 0.9 % saline and medium (1:1) following serial dilutions in RPMI 1640. The drug exposure period was 24 h. After this, supernatants were removed by suction, and each well was washed with PBS. A further 72 h was allowed for the cells to recover in drug-free medium at 310 K. The SRB assay was used to determine cell viability.[39] Absorbance measurements of the solubilized dye (on a BioRad iMark microplate reader using a 470 nm filter) allowed the determination of viable treated cells relative to untreated controls using the inflection point of a dose–response graph. IC_50_ values (concentrations that caused 50 % cell growth inhibition) were determined as duplicates of triplicate readings in two independent sets of experiments and their standard deviations were calculated.

**IC_50_ modulation experiments**: Experiments to investigate the effect of co-administration of artemisinin and 3CA were carried out as described above, with the following modifications: cells were pre-incubated in drug-free medium for 48 h at 310 K, before adding artemisinin together with 3CA. To prepare stock solutions of the drug, the solid artemisinin was dissolved first in 5 % DMSO and then diluted in a 1:1 mixture of 0.9 % saline and the cell culture medium. This stock was further diluted using RPMI 1640 until working concentrations were achieved. Separately, a stock solution of 3CA was prepared in a similar manner. Both solutions were added to each well independently, but within 5 min of each other. Once again the drug exposure time was 24 h and the drug-free recovery time was 72 h. The SRB assay was used to determine cell viability. IC_50_ values were determined as duplicates of triplicates in two independent sets of experiments and their standard deviations were calculated.
